# Bridging cholinergic signalling and inflammation in schizophrenia

**DOI:** 10.1038/s41537-024-00472-2

**Published:** 2024-05-11

**Authors:** Christine N. Metz, Michael Brines, Valentin A. Pavlov

**Affiliations:** 1 The Feinstein Institutes for Medical Research, Northwell Health, Manhasset, NY 11030 USA; 2 Zucker School of Medicine at Hofstra/Northwell, Hempstead, NY 11550 USA; 3 Elmezzi Graduate School of Molecular Medicine, 350 Community Drive, Manhasset, NY 11030 USA

**Keywords:** Schizophrenia, Diseases, Psychiatric disorders

Schizophrenia is a severe multifactorial mental illness and a leading cause of global disability^1,2^. Current options for pharmacological treatment of schizophrenia are almost entirely based on blocking brain dopamine D2 receptor signalling^1^. However, the efficacy and tolerability of therapeutics targeting this mechanism are limited and unsatisfactory^1^. This dictates the need for developing better treatments, which may come from new insights into the pathophysiology of schizophrenia.

Peripheral inflammation is implicated in the pathophysiology of schizophrenia, and provides a therapeutic target, but its intricate relationship with brain alterations were uncertain^3–6^. A recent study, utilizing an advanced semi-supervised machine learning approach revealed a link between distinct inflammatory profiles and brain alterations in schizophrenia patients^7^. Of note, the most widespread brain neuroanatomical impact was observed in a group of patients with increased circulating levels of the cytokines IL-6 and IL-8 and designated as a classic inflammation cluster^7^. These findings suggested that patients in an inflammatory cluster may benefit the most from targeted anti-inflammatory treatments.

There are many anti-inflammatory modalities, but what about a treatment, with demonstrated but different from the mainstream dopamine D2 receptor-mediated antipsychotic efficacy, which in addition has anti-inflammatory effects? Accumulating evidence from preclinical, clinical, post-mortem, brain-imaging and pharmacological studies reveals an involvement of brain cholinergic signalling in the pathophysiology and treatment of schizophrenia^8^. Preclinical evidence has demonstrated the antipsychotic-like effects of xanomeline, a centrally acting M1/M4 muscarinic acetylcholine receptor agonist and the key role of brain muscarinic receptors in mediating these effects^9–11^. These preclinical studies in rodents and in non-human primates^12^ indicated that the cholinergic compound xanomeline may be a useful treatment for psychosis. The efficacy of xanomeline monotherapy in improving positive symptoms and cognition in 20 individuals with schizophrenia was among the first evidence supporting this notion^8,11^. Recently, in a double blind, placebo controlled trial with 182 patients, xanomeline used in combination with the peripherally restricted muscarinic receptor antagonist trospium (for mitigating undesirable peripheral cholinergic effects) significantly decreased positive and negative symptoms in acutely psychotic patients^13^. Currently, there is a substantial interest in further developing xanomeline and other centrally acting M1/M4 muscarinic acetylcholine receptor agonists, including positive allosteric modulators such as emraclidine (CVL-231) in the treatment of this disorder^1,11^.

Brain muscarinic receptor mediated cholinergic signalling also regulates peripheral inflammation^14–17^. Of note, xanomeline significantly suppresses pro-inflammatory cytokine levels (including IL-6) in animals with aberrant inflammation^15^. These anti-inflammatory effects of xanomeline are mediated through brain muscarinic acetylcholine receptor signalling, because they are abrogated in animals pretreated with the centrally acting muscarinic receptor antagonist atropine sulfate, but not by the peripherally restricted muscarinic receptor antagonist atropine methyl nitrate^15^. Neural signalling through the efferent vagus and splenic nerve in a major physiological mechanism termed *the inflammatory reflex*^18–20^ provides an important brain to periphery communication channel further mediating xanomeline anti-inflammatory effects^15^.

Considering these findings from independent lines of research, one may now ask whether the potent anti-inflammatory activity of xanomeline contributes to its robust beneficial effects in schizophrenia. The recent work^7^ provided a platform that can be applied in future trials to address this question by examining whether schizophrenia patients with increased peripheral inflammation specifically benefit from xanomeline treatment. An important consideration in such future studies will be mitigating the possible peripheral cholinergic side effects of xanomeline treatment, including constipation, nausea, dry mouth, dyspepsia, and vomiting. As recently shown, this can be successfully achieved by giving xanomeline in combination with the peripherally restricted muscarinic receptor antagonist trospium without compromising xanomeline beneficial antipsychotic effects^13^. As the anti-inflammatory effects of xanomeline are mediated through brain muscarinic receptor cholinergic signalling and not through peripheral muscarinic receptors^15^ adding trospium is not expected to interfere with xanomeline anti-inflammatory efficacy.

In addition to xanomeline, other centrally acting compounds, including the acetylcholinesterase inhibitor galantamine, exert significant anti-inflammatory effects, which are mediated through brain muscarinic receptors and linked to neural signalling along the efferent vagus nerve arm of the inflammatory reflex^16,21^. Galantamine is a cholinergic drug clinically approved for treating cognitive deterioration in patients with Alzheimer’s disease^22,23^. This cholinergic drug is well tolerated when the drug dose is gradually escalated^22–24^. The robust anti-inflammatory and beneficial metabolic activities of galantamine have been demonstrated in preclinical settings of numerous inflammatory conditions^22^ and in patients with the metabolic syndrome^24^. The inflammatory reflex can be also activated by electrical vagus nerve stimulation^17^. The anti-inflammatory effects of vagus nerve stimulation are mediated through the alpha7 nicotinic acetylcholine receptor expressed on macrophages and other immune cells^25^, but not through peripheral muscarinic receptors^14^. The anti-inflammatory and disease alleviating effects of vagus nerve stimulation have been shown in many inflammatory conditions in rodents^17^. Using implanted bioelectronic devices or non-invasive approaches, vagus nerve stimulation has been also successfully explored in treating patients with chronic inflammatory conditions, including inflammatory bowel disease, rheumatoid arthritis, and other disorders^17^.

It might be suggested that, in addition to xanomeline, cholinergic modalities such as galantamine or vagus nerve stimulation could be utilized in treating schizophrenia patients with increased inflammation.
